# In-Service Delaminations in FRP Structures under Operational Loading Conditions: Are Current Fracture Testing and Analysis on Coupons Sufficient for Capturing the Essential Effects for Reliable Predictions?

**DOI:** 10.3390/ma16010248

**Published:** 2022-12-27

**Authors:** Andreas J. Brunner, René Alderliesten, John-Alan Pascoe

**Affiliations:** 1 Empa, Swiss Federal Laboratories for Materials Science and Technology, Laboratory for Mechanical Systems Engineering, CH-8600 Dübendorf, Switzerland; 2 Department of Aerospace Structures & Materials, Faculty of Aerospace Engineering, Delft University of Technology, 2629 HS Delft, The Netherlands

**Keywords:** fibre-reinforced polymer-matrix composites, fatigue fracture tests, planar delamination initiation and propagation, multidirectional laminate lay-up, variable environmental exposure, predictive modelling

## Abstract

Quasi-static or cyclic loading of an artificial starter crack in unidirectionally fibre-reinforced composite test coupons yields fracture mechanics data—the toughness or strain-energy release rate (labelled G)—for characterising delamination initiation and propagation. Thus far, the reproducibility of these tests is typically between 10 and 20%. However, differences in the size and possibly the shape, but also in the fibre lay-up, between test coupons and components or structures raise additional questions: Is G from a coupon test a suitable parameter for describing the behaviour of delaminations in composite structures? Can planar, two-dimensional, delamination propagation in composite plates or shells be properly predicted from essentially one-dimensional propagation in coupons? How does fibre bridging in unidirectionally reinforced test coupons relate to delamination propagation in multidirectional lay-ups of components and structures? How can multiple, localised delaminations—often created by impact in composite structures—and their interaction under service loads with constant or variable amplitudes be accounted for? Does planar delamination propagation depend on laminate thickness, thickness variation or the overall shape of the structure? How does exposure to different, variable service environments affect delamination initiation and propagation? Is the microscopic and mesoscopic morphology of FRP composite structures sufficiently understood for accurate predictive modelling and simulation of delamination behaviour? This contribution will examine selected issues and discuss the consequences for test development and analysis. The discussion indicates that current coupon testing and analysis are unlikely to provide the data for reliable long-term predictions of delamination behaviour in FRP composite structures. The attempts to make the building block design methodology for composite structures more efficient via combinations of experiments and related modelling look promising, but models require input data with low scatter and, even more importantly, insight into the physics of the microscopic damage processes yielding delamination initiation and propagation.

## 1. Introduction

There have been extensive efforts made which aim to better understand the fracture of fibre-reinforced polymer-matrix (FRP) composites, as well as the development and standardisation of fracture test methods, both under quasi-static and cyclic fatigue loading [1,2,3]. Such test data are useful for, e.g., quality control, material selection and assessing process or material modifications with respect to delamination resistance. However, the most promising application is the determination of fatigue fracture mechanics-based design limits for FRP composite structures and elements [4]. These are expected to allow significant reductions in structural weight in damage-tolerant designs compared with conventional designs based on the so-called “no growth” design approach [5,6,7].

The development of standardised procedures for the fatigue fracture testing of FRP composites started in the 1980s and is still ongoing. ASTM D6115 applies to the measurement of fatigue delamination onset under tensile opening Mode I loading, but excludes delamination propagation. Standard tests for measuring stable fatigue delamination propagation in FRP composites are still currently unavailable. The efforts to develop such standards and open questions posed by these activities have been summarised and reviewed, e.g., in [1,8,9]. Pending the solution of several challenging issues, the successful development of such standards is still some way off and requires significant further testing effort. The major points discussed in this contribution are: (1) The development of a procedure which can successfully account for fibre bridging, which is frequently observed in the unidirectionally fibre reinforced test specimens recommended by the standards, but which is less important in woven lay-ups (see, e.g., [10]) or quasi-isotropic lay-ups [11]. (2) The differences between two-dimensional delamination propagation in plate- or shell-like structural elements versus essentially one-dimensional propagation in beam specimens (see, e.g., [12,13,14]). (3) How to approach the complications posed by multiple delaminations generated by impact or resulting from processing, e.g., by residual stresses, or often observed in multidirectional laminate lay-ups (see, e.g., [15,16,17,18,19]). (4) Approaches to reducing the 10–20% scatter typically observed in the repeatability and reproducibility of quasi-static and fatigue fracture test data, as well as reducing the differences between experiments and the modelling of structural behaviour, which can be up to 50%. The scatter in the experimental test results has been shown to originate largely from processing and test operator actions (see, e.g., [20,21]). (5) The development of experimental approaches and extrapolation procedures capable of predicting the long-term effects of variable service environments on delamination initiation and propagation. (6) The relationship between the toughness of neat polymers and that of composites using these polymers as a matrix. Finally, in a brief outlook, selected promising approaches for some of the issues are noted.

## 2. Materials and Methodology

**Figure 1:**
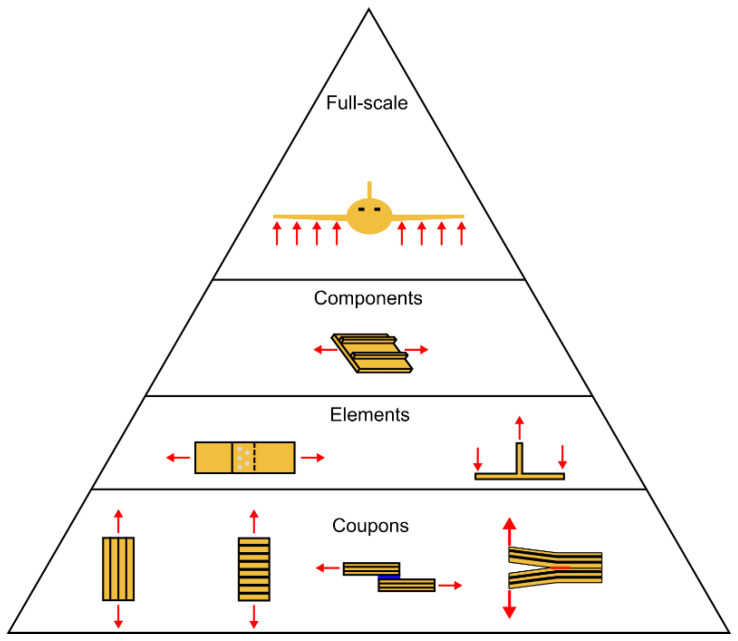
Schematic example of a test pyramid for a composite aircraft. In practice, additional interme-diate levels may be defined (see, for example, [22]); the red arrows schematically indicate the direction of the applied loads for the different test cases (the magnitude of the arrows does not necessarily correlate with the applied loads).

The limited availability of test standards for the fatigue fracture of FRP composites was noted in the previous section. However, even when standard test methods become available, fracture properties measured in coupon tests are not always sufficient to understand the behaviour of full-scale structures. This is due, for example, to size effects, manufacturing defects or tolerances, or the complex stress concentrations produced by the geometrical features of a full-scale structure. These effects are not present in small coupons and cannot always be easily captured by modelling. In CFRP composites, there is the additional challenge whereby differences in lay-up can produce damage mechanisms not seen in smaller-scale coupons. To accommodate these issues, structural development often follows a so-called building block or test pyramid approach (Figure 1). This approach is especially important in weight-critical sectors such as aerospace, where the application of large safety factors would impose unacceptable weight penalties. In the building block approach, a large number of coupons are tested to generate material data, followed by a smaller number of specimens representing structural elements (e.g., a pin-loaded hole or a single-lap adhesive joint), etc., leading to a very small number of full-scale tests. The idea of this approach is that the coupon testing can generate necessary material data and insight, which can then inform the higher-level testing. One example is the use of knockdown factors to account for environmental effects, as it would be very expensive to have to test components or full-scale structures under all possible environmental conditions.

To limit costs, the testing at higher levels of the pyramid should be minimised, and this can be achieved by learning as much as possible from the lower-level (coupon and element) tests, and then, generalising the results to predict the behaviour of larger-scale components. The next sections will highlight the key knowledge gaps that currently prevent such generalisation and, therefore, necessitate additional testing at higher levels of the pyramid.

Throughout this paper, many different results from the literature will be discussed. For the full details on the materials and test methods used in each case, the reader is referred to the referenced literature.

## 3. Relevant Issues and Discussion

### 3.1. Accounting for Fibre Bridging Effects

#### 3.1.1. The Fibre Bridging Phenomenon

With unidirectionally fibre-reinforced specimens tested in Mode I (the tensile opening mode), fibres may bridge the crack as they remain attached to both adherends. This phenomenon is generally attributed to nesting of the unidirectional fibres of two adjacent plies ahead of the artificial starter crack [23]. Fibre bridging acts as crack-tip shielding, which increases the quasi-static fracture toughness, yielding a progressively increasing R-curve (R stands for “resistance”) until a plateau is reached [24,25]. Similarly, it increases the energy release rate (expressed as G_max_, ΔG, or (Δ√G)^2^) required to achieve a certain crack growth rate [26]. These changes—which can be observed as translation and rotation of the so-called Paris curves [27]—have contributed to the lack of consensus on how to experimentally evaluate the behaviour, which would be necessary for standardisation.

One of the key issues identified as potentially leading to the above problem is related to the pre-cracking of the test specimens prior to fatigue fracture testing, a procedure essentially similar to quasi-static testing [28,29]. The pre-crack lengths obtained using the pre-cracking procedures may vary by a few millimetres, and this has been observed to have a significant effect on the position of the measured Paris curve [4,30]. This was initially interpreted as being due to significant scatter, but it is more likely related to the generation of different amounts of fibre bridging [26]. A longer pre-crack has the potential to possess a longer fibre-bridged zone. As discussed by Jones et al. [31], for design, an ‘upper bound’ curve is required, which provides the worst case (fastest crack growth rate) at a given applied load, taking the scatter into account. This essentially requires a zero pre-crack length, (associated with zero initial fibre bridging), which is impossible, as it, depending on the thickness of the starter film, may constitute a blunt starter crack tip. At the same time, every millimetre of pre-cracking yields a more non-conservative curve for a composite showing a rising R-curve.

Various authors have attempted to evaluate the amount of fibre bridging through numerical simulation [32,33,34]. They have demonstrated that this procedure can provide an understanding of the bridging fibres’ contribution. However, this research has opened up further discussion on the suitability of linear elastic fracture mechanics to evaluate this problem. It has been suggested that non-linear, and specifically elastic plastic fracture mechanics, should be adopted to establish the J-integral, rather than using the strain energy release rate G [35]. Additionally, one could argue that the test results in this case are only as good as the model adopted in the simulation of the structural behaviour.

#### 3.1.2. Evaluating the Zero-Bridging Curve

**Figure 2:**
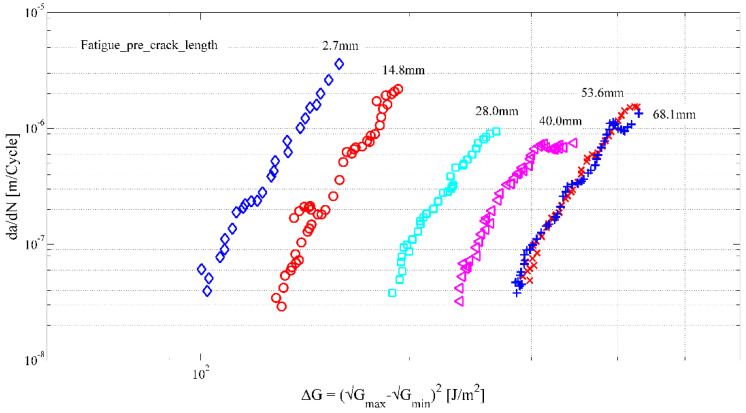
Paris curves for delamination propagation from six test sequences on a single DCB specimen illustrating the influence of pre-crack length and fibre bridging length [36].

When a mode I fatigue delamination propagation test is performed in displacement-controlled conditions, the strain energy release rate decreases with decreasing force. This implies that the Paris curve is generated from a maximum value towards retardation in the threshold region [36]. Continuing the test beyond this point by increasing the applied displacement essentially repeats the procedure, yielding a second curve, positioned to the right of the first curve. Repeating this procedure multiple times yields multiple Paris curves, as illustrated in Figure 2, until the curves start to overlap. This overlap in the Paris curves corresponds to the plateau in the quasi-static R-curve; the strain energy release rate required for a certain crack growth rate remains constant beyond that point [36].

Through the regression of a non-linear surface through all the da/dN-(Δ√G)^2^ data obtained from these multiple curves, one can find a single expression describing all curves:

(1) $$Log\left(\Delta \sqrt{G}\right)=C_{0}+C_{1}\left(a-a_{0}\right)+C_{2}Log\left(\frac{da}{dN}\right)+C_{3}{\left(a-a_{0}\right)}^{2}+C_{4}{\left[Log\left(\frac{da}{dN}\right)\right]}^{2}$$

This approach has the advantage that scatter is no longer attributed to the variability in the positioning of the Paris curves, as they are inherently described by the non-linear regression. Instead, intrinsic scatter can be quantified by taking the maximum error between each data point and the nearest point on the regression surface. The relationship for the curve describing the delamination resistance excluding fibre bridging is obtained by setting the terms (a-a_0_) to zero [27].

Several other methods have been proposed to derive an upper bound Paris curve which excludes fibre bridging. One method to evaluate the strain energy release rate range in the presence of fibre bridging was proposed in Ref. [37], which was evaluated with equivalent test data against Equation (1) to yield approximately similar upper bound curves [36]. Similarly, a variant of the Hartman–Schijve relationship yields such an upper bound, through incorporating, in essence, similar fracture toughness and threshold parameters, but in a different formulation [31,38]. A method requiring different fatigue delamination experiments has been proposed by Hojo and Aoki [39], which is based on constant G_max_ rather than constant displacements.

Where all the studies above focused on fatigue delamination under constant amplitude displacement loading, recent variable amplitude loading testing has demonstrated that predictions might lead to non-conservatism if transient phenomena related to fibre bridging are not considered [40,41,42].

### 3.2. Two-Dimensional Delamination Propagation versus That in Standard Beam Specimens

**Figure 3:**
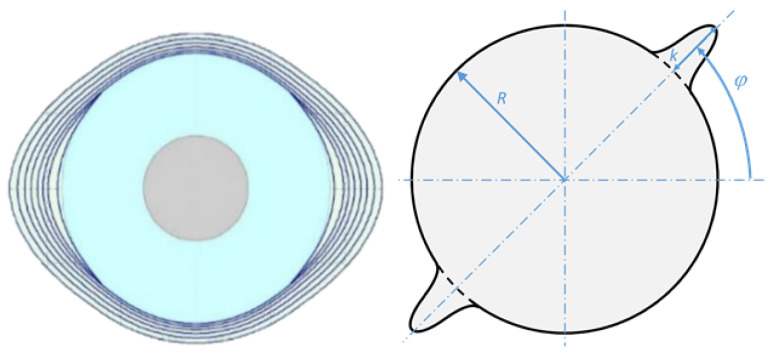
Illustration of planar delamination propagation (left) and propagation in a single transverse direction (right) [45].

In-service delaminations generally develop in a planar fashion, rather than in one direction, as tested in the standardised delamination tests. In planar fatigue delamination, more variation in growth patterns might occur, ranging from entirely planar to predominantly transverse (see Figure 3). These variations can be attributed to the loading mode applied [43] and the magnitude of loading, which is known to affect the resistance of the material to fracture [44].

To predict planar delamination propagation, essentially, the interplay between multiple effects must be considered. The first effect in the description of delamination resistance is fibre orientation at both sides of the interface. Where unidirectional tests only characterise one specific orientation, the circumference of a planar delamination experiences a full range of orientations relative to the propagation direction. In addition, the shape of the delamination, combined with the orthotropy of the laminate at both sides of the interface, imposes a transverse constraint on the local crack tip. This can be incorporated through defining the strain energy density at the crack contour [14]. The fibre bridging discussed before is expected to reveal itself differently in planar problems due to different fibre orientations, which is a subject still to be evaluated [46,47].

**Figure 4:**
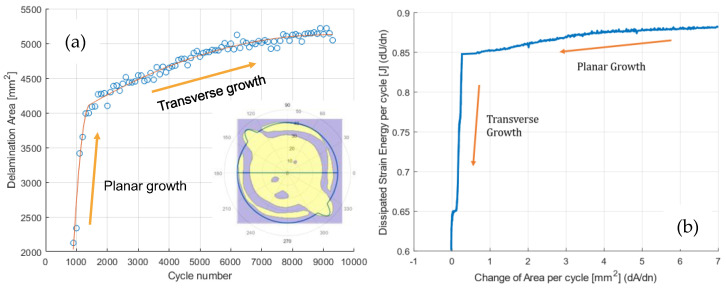
Fatigue delamination area propagation curve exhibiting a transition from planar to transverse growth (**a**) and the dissipated strain energy associated with the areal growth (**b**) [14].

Planar fatigue delamination tests in carbon fibre-reinforced polymer composites have illustrated that the initial planar growth can be overtaken by transverse growth (see Figure 4), depending on the amount of work (strain energy) offered, relative to the total delamination resistance along the increasing crack contour [48]. This transition in delamination resistance, illustrated in Figure 4b, is currently not well considered in the yet-to-be-standardised fatigue delamination tests [14].

### 3.3. Multidirectional Laminates and Multiple Delaminations in Composite Testing

The development of quasi-static and fatigue delamination propagation tests made use of unidirectional laminates, with the same fibre orientation on either side of the delaminating interface. This configuration is not very representative of multidirectional laminates, where delaminations usually occur at the interfaces between plies with different fibre orientations. Furthermore, in many cases, delaminations will not just initiate in a single interface, but in multiple interfaces at once, for example, in the case of impact damage. The effect of fibre orientation and the presence of other delaminations on delamination propagation under fatigue loading has not received much attention so far.

A summary of investigations into the fibre orientation effect on quasi-static loading has been recently provided by Blondeau et al. [24]. They conclude that the fibre orientation does not affect the initiation fracture toughness, but it does affect the propagation fracture toughness and R-curve behaviour. This is because initiation occurs in a small resin-rich region, relatively unaffected by the fibres, whereas the subsequent crack propagation is strongly dependent on the lay-up. In many cases, crack migration occurs, which increases the (apparent) fracture toughness, at least at the macroscopic level (the resin fracture toughness as a material property is unlikely to be affected). Depending on the lay-up, the migration can cause the crack to progressively move away from the initial interface [49] or oscillatory fracture behaviour can be observed [50].

Under fatigue loading, Singh and Greenhalgh [51] provided a detailed description of microcracking and crack migration mechanisms in a 0°//90° interface. Yao et al. [26] found a higher fatigue threshold for a 45°//45° interface compared to a 0°//0° interface, while Peng et al. [52] reported that the fatigue thresholds of a +45°//−45° interface, a 0°//5° interface and a 90°//90° interface were similar if normalised by the quasi-static fracture toughness. Other researchers have investigated fatigue delamination propagation in multi-directional interfaces, but without comparing the behaviour to a unidirectional interface [53,54,55,56]. Preliminary research reported by van der Panne [46] suggests that the Paris curve shifts that result from fibre bridging (see Section 3.1) are affected by the fibre orientation.

More data on fatigue-driven delamination propagation in multi-directional interfaces is needed, but a valid conceptual approach should also be carefully considered. Describing the crack propagation behaviour in detail would require full modelling of delamination migration, which could be prohibitively expensive (in terms of both computation and model set-up time) for large-scale structures. For application purposes, a more fruitful approach could be to assign the interface effective delamination resistance, which is a function of the fibre orientations on each side of the interface. The effective resistance value could then be determined from micro-mechanical modelling and/or experiments.

The occurrence of multiple delaminations has been experimentally observed [11,15,16,17,18], even in unidirectional DCB specimens [17,57]. For Mode I DCB specimens. Khudiakova et al. [17] made use of a damage parameter introduced by Brunner et al. [58] to capture the effect of multiple delaminations. Goutianos and Sørensen [59] developed a J-integral-based model to predict the propagation of multiple delaminations under quasi-static loading. In both cases, only the interaction between two delaminations was investigated. The work of Yang [60] suggests that the quasi-static behaviour in compression after impact depends on the configuration of delaminations at all interfaces, and one can also expect this to hold for fatigue fracture. The intentional initiation and propagation of multiple delaminations has been shown to yield a beneficial toughening effect in a numerical study [61], as well as in experiments [62]. Therefore, more research is needed on interactions between multiple delaminations and how to efficiently model these during fatigue- and damage-tolerance analyses of composite structures. In principle, it should be possible to adapt existing numerical models for this purpose. Models for compression after impact have been shown to be capable of dealing with the presence of many delaminations (see, e.g., [60,63,64]). The issue here for fatigue delamination propagation is to keep the computational cost manageable.

### 3.4. Scatter and Sources of Scatter in Testing and Modelling

The scatter observed in quasi-static or fatigue fracture test development is usually determined in so-called ‘round robin’ tests, where several test laboratories perform the fracture tests according to a given specification (the ‘protocol’) on the same material, ideally from the same batch. Repeatability is then assessed according to the in-laboratory scatter, e.g., calculating the standard deviation of the fracture toughness data from the sample (often five specimens per laboratory) for each laboratory separately. Reproducibility, i.e., inter-laboratory scatter, then represents the scatter among the data from all the laboratories pooled. Round robin results with scatter for quasi-static Mode I delamination resistance have been published, e.g., in References [65,66,67] and for quasi-static Mode II, e.g., in References [20,65]. For Mode I fatigue delamination resistance, the repeatability and reproducibility have been discussed, e.g., by Stelzer et al. [68,69]; for Mode II fatigue delamination resistance, selected data from literature are compared in Figure 8.6 on p. 212 of Reference [9]. The repeatability for quasi-static Mode I tests, as determined by O’Brien and Martin [67], amounts to between 7% and 17% for CFRP epoxy laminates, and reproducibility to between 12% and 19%. For CFRP PEEK, Mode I repeatability amounts to between 10% and 13%, and reproducibility to between 8% and 18% in the first round robin, and in the second round robin, between 8% and 14% (repeatability) and 8% and 18% (reproducibility). It is noteworthy, however, that in this round robin analysis, not all specimens conformed to the criteria defined in the final standard procedure. For example, some of the specimens tested had a thicker starter film (25 micrometres instead of 13 micrometres). A round robin investigating quasi-static Mode II with three different test set-ups [20] reported repeatability between 3% and 23%, depending on the type of test configuration, and reproducibility between 14% and 51%, again, depending on the test configuration. If standard test specimen design and test procedures are followed, repeatability and reproducibility for quasi-static Mode I and Mode II, with very few exceptions, are roughly between 10% and 20%. It can be speculated that larger scatter may partly be caused by a lack of test experience, e.g., as in some of the first round robins cited above. For a discussion of the sources of scatter, it is useful to estimate the experimental variability resulting from the measurement resolution required by the standards. As discussed by Brunner [21], load, displacement and delamination length values each have to be determined with about a 1% precision. An error estimate based on Gaussian error propagation then yields about 4–5% repeatability for a set of delamination resistance measurements. For single data points in so-called Paris-plots for data from Mode I fatigue fracture, calculated essentially from the same measurements, a similar error will apply. The assessment of scatter in fatigue fracture thresholds and delamination propagation curves is somewhat more complex than that in single-point fracture initiation data from quasi-static fracture. This is discussed in detail in References [4,68,69], specifically considering extrapolation of experimental data to the threshold region, defined by a limit of the average delamination propagation per cycle and the respective scatter.

Sources of scatter have been identified as coming mainly from the variability in material properties, e.g., manual processing, on one hand, and from test operator actions in test set-up and data analysis, on the other [21]. With respect to material variability, it is essential that test coupons are representative of the material quality for elements, components and structures. Otherwise, the design values derived from test coupons may overestimate the delamination resistance of components or structures.

An example of the scatter due to manual analysis is the repeatability of initiation points from a single load displacement curve published by Davies [70]. The non-linear point determination by 36 people yielded a standard deviation of 4.8%, and the initiation point defined by a 5%-increase in compliance yielded a standard deviation of 3.6%. This was interpreted to indicate that such effects are likely to contribute significantly to an overall reproducibility of 10% or more for quasi-static Mode I tests.

**Figure 5:**
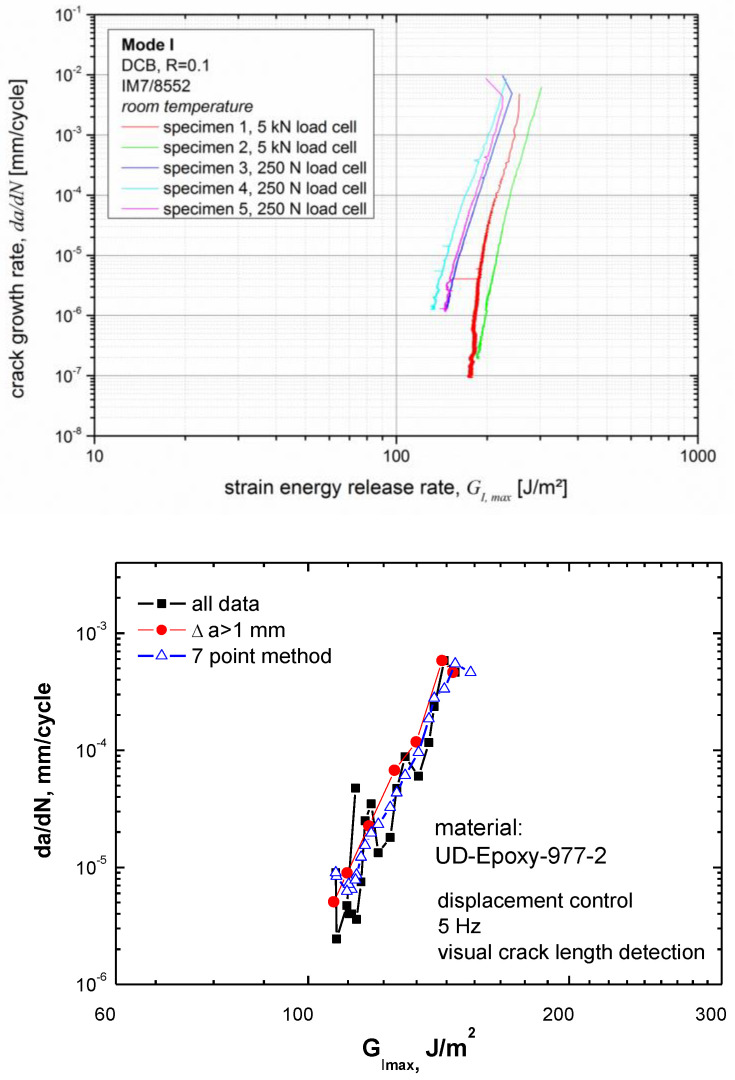
Graphs illustrating scatter in Mode I fatigue fracture testing: (top) for the CFRP laminate (IM7/8552), due to differences in test set-up, except for the capacity range of the load cell (5 kN versus 250 N), the set-up is identical; (bottom) for the CFRP laminate (IM7/977-2), from data analysis, the full data set from visual delamination length observation (black squares) is reduced by showing only data with at least a 1 mm delamination length increase (red circles), and by fitting using the seven-point method (blue triangles) according to [72].

Selected additional examples of scatter from the test set-up and data analysis are illustrated in Figure 5. The first graph indicates that the chosen measurement range of the load cell can have a significant effect on the position of the fatigue delamination propagation curves. All curves measured with a load cell range of 0–250 N appear at lower G_Imax_ values than those from the same material and the identical test set-up with a 5 kN load cell. It is important to note that this difference is not caused by variation in the initial delamination lengths. Different pre-cracking lengths for starting Mode I fatigue fracture have previously been shown to induce a similar shift in the delamination propagation curves (see, e.g., Figure 3 on p. 2682 in Ref. [71]). The second graph in Figure 5 compares a propagation curve obtained from visual delamination length data with a smoothed curve plotted with data points only for delamination lengths that were at least 1 mm apart and with a curve smoothed using the seven-point polynomial fitting procedure described in ASTM E647 [72]. Such smoothing procedures will reduce the scatter in threshold values determined from extrapolation of the experimental propagation curves to a nominal da/dN threshold value (e.g., 10^−8^ mm per cycle), for which the expected number of service life cycles will not yield a critical delamination length. Extrapolations taking the full scatter in the curve from the raw data into account will usually yield a more conservative, lower threshold value.

Approaches to improving repeatability and reproducibility in testing, hence, are highly automated material manufacturing for consistent laminate quality and digital tools for data analysis eliminating subjective operator decisions [21].

Scatter at the structural level is affected by the observation that fatigue fracture in this case does not just involve damage from matrix cracking, i.e., delamination propagation and intralaminar cracking, but also from fibre–matrix debonding and fibre failure. Furthermore, these damage mechanisms will interact. To avoid this complexity, many researchers have adopted finite element-based approaches such as continuum damage mechanics (CDM). In these approaches, element properties are progressively degraded, rather than explicitly modelling the physical damage (see, e.g., Refs. [73,74,75,76]). Recently, the US Air Force Research Laboratory benchmarked the performance of seven different models against experimental data [77]. The average errors recorded for the blind prediction of residual strength are shown in Table 1.

Overall, on average, the blind predictions of residual strength and stiffness differed from the test data by 42%. After recalibration of the models, this improved to 18%. One of the reasons identified for the poor performance was the inability of the models to represent the damage micro-mechanics, and in particular, the fatigue delamination propagation. Because the models do not explicitly include this, they are unable to account for changes in damage mechanisms between different lay-ups. In this case, for the blind predictions, the models were calibrated with coupon data for 0^0^, 90^0^ and ±45^0^ lay-ups. The prediction error was much larger for the [60/0/-60]_3S_ lay-up than for the [0/45/90/-45]_2S_. It seems likely that this difference in performance relates to the limited ability of these models to generalise beyond the lay-ups used to generate the input material data. In practice, it is undesirable to have to test each different lay-up that will be used in a design. Thus, further work is needed to understand how to correctly model the damage (micro)mechanisms, such that data from a small set of coupons can be generalised to accurately predict the behaviour of a laminate with an arbitrary lay-up.

Regarding the analysis of fatigue delamination propagation in composite elements and components during design, Jones et al. [4] conclude that (cite) “*The experimental data also reveals that DCB fatigue test results usually show a great deal of scatter, which may arise from fibre-bridging developing during the test. It is therefore very difficult to determine a meaningful ‘average’ delamination growth curve. The same comments are true with respect to determining a valid value of the fatigue threshold, below which no significant FCG* [Fatigue Crack Growth] *occurs. Thus, a methodology is needed for estimating a valid upper-bound curve which encompasses all the experimental data and provides a conservative FCG curve and which is representative of a composite laminate exhibiting no, or only very little, retardation under fatigue loading. Such a valid, upper-bound curve can then [be] employed for (a) the characterisation and comparison of composite materials, (b) a ‘no growth’ design, (c) for assessing if a delamination, that is found in an in-service aircraft, will grow and (d) the design and lifing of in-service composite aircraft structures where material allowable properties have to be inputted into a delamination growth analysis*”. The upper-bound curves for design obtained from the Hartman–Schijve approach as proposed in Reference [4], of course, strongly depend on the experimental scatter in the coupon tests providing the data on delamination propagation behaviour in CFRP laminates.

When constructing such upper-bound curves, it is also important to distinguish between the scatter caused by the test set-up and execution and scatter due to ‘natural’ material defects or the limits of manufacturing quality control. While scatter due to the test set-up is not representative of the actual material behaviour, the latter form of scatter—which one could call intrinsic scatter—is. Additionally, in full-scale structures, there will be natural defects and manufacturing variability. Therefore, it is important to take these sources of scatter into account during design. This requires accurate determination of their magnitude during material characterisation. In short, while all efforts should be taken to reduce scatter due to experimental artefacts during material characterisation, intrinsic scatter should be preserved and measured, to ensure the test results are representative of the full-scale structure.

### 3.5. Issues with Prediction of FRP Delamination Resistance Behaviour in Service Environments

For certain applications of neat polymers, prediction methods have been developed that yield reliable service life data for components made from them. One example is the standard extrapolation method for thermoplastic pipes that provides reliable long-term predictions [78]. Alternative fracture mechanics-based approaches for such predictions, with specimens that use less material than the pipe segment tests, are discussed, e.g., in Refs [79,80]. Recently, fracture mechanics-based life-time investigations for polymer pipes have been shown to achieve reasonable predictions over 50 years at least, and possibly up to 100 years [81]. This clearly shows the potential of facture mechanics-based predictions of service life; however, of course, the development and the proof of applicability of such a methodology to continuous CFRP composites for aerospace is still lacking, even though expected service lives are typically less than 50 years.

A method used to determine a so-called master curve for the property prediction of polymers is the time–temperature superposition. This method has recently been applied to accelerated testing for static tensile and fatigue strength, as well as the creep of CFRP [82], but no comparable method yet exists for the delamination resistance of CFRP. The creep behaviour of unreinforced thermoplastic polymers from a master curve has also been obtained using a stepped iso-stress method (SSM), as discussed by Hadid et al. [83]. The authors conclude that (cite) “*A smooth creep master curve has been obtained. The obtained master curves by the SSM technique and the classical TSSP* [time- -stress superposition principle] *method are consistent. This result proofs the robustness of the SSM technique in the construction of the master curve*”.

This stepped iso-stress method has also been applied to the creep and creep rupture of CFRP by Tanks et al. [84] and the authors conclude that the stepped iso-stress method (cite) “… *employs a load-stepping approach, typically with three to five steps for a single specimen resulting in creep-rupture. Theoretical analysis of the experimental method (previously lacking) is discussed and modifications to the testing and analysis protocol are shown to improve validity of the master curves compared to conventional creep curves and traditional TTSSP* [time–temperature–stress superposition] *creep master curves at room temperature. Finally, three failure criteria are compared for predicting creep-rupture of CFRP laminates based on the accelerated testing method*”. They add that (cite) “*The data analysis procedure for the SSM is similar to the traditional TSSP* [time–stress superposition] *approach, except that one specimen is used for all stress levels instead of individual specimens; this requires a rescaling step to account for stress history before applying the time-stress shift factor to construct the master curve*”. The creep rupture test analysis presented in Ref. [84], however, is not based on fracture mechanics, and whether developing an analogous fracture mechanics approach to fatigue delamination propagation is feasible remains an open question.

A study of fatigue damage and delaminations induced by tensile and bending loads in aeronautical CFRP samples, i.e., in laboratory-scale plates and open-hole tensile specimens, first monitored damage development under high cycle fatigue with ultrasonic and thermographic full-field inspection [85]. The damage locations found were then investigated via FEM for static failure-zone identification and these showed a reasonable correlation with the experimental high cycle fatigue (HCF) results. The authors note that (cite) “*In bending test case, static behaviour is well correlated to FEM model and successively estimation of the progressive fatigue damage evolution is possible when lower load is applied for HCF loading conditions. Fatigue experimental results highlights general data variation in damage progress, but analysing different damage conditions of specimens, good coherence between experimental inspection and stiffness variation is observed, anticipated by continuous and early damage nucleation identified through thermal and ultrasonic monitoring*”.

This indicates the potential of combining non-destructive inspection for damage identification, followed by modelling for the prediction of delamination initiation and propagation in CFRP under mechanical loads. One question related to this methodology is whether it is applicable to larger-scale parts and components, e.g., in the building block approach for structural design [86]. An alternative to the building block approach combining experiments and numerical modelling, so-called ‘smarter testing’, is discussed in more detail below. However, the effects of arbitrary mechanical load spectra combined with varying types of environmental exposure (the latter is discussed below in detail) may still be too complex for reliable prediction via this ‘smarter testing’ approach. An important aspect in this, besides the load spectra, is the operational environment and its variation with service duration.

The ambient operating conditions and their variation can play a significant role in determining damage accumulation, and hence, the service life of CFRP components and structures. Components or structures made from different materials are known to age with time, limiting their service life, and this applies to FRP composites, as well [87,88,89]. Under varying types of loads and service environments, properties may tend to degrade at different rates (see, e.g., Reference [90] for a discussion of changing environmental condition effects on FRP building materials). The authors conclude that (cite) “*The minimum SL* [service life] *of BEMs* [building envelope materials] *is either 25 or 50 years, depending on the building type, and yet no RSLV* [reference service life values] *have been established, or SLPs* [service life predictions] *made based on experimental work. The way forward to SLPs that account for climate change is thus clear: establish reference values based on accelerated aging methods that integrate climatic conditions expected in 25 to 50 years. This type of work will require significant computer-modelling efforts, both in terms of climatic models, which is well underway, and material degradation models, which must be able to integrate all the degradation factors, including UV radiation, moisture, temperature conditions, mechanical stresses and biological factors*”.

For aerospace grade composites, the current status of the prediction of service life is comparable to that of the building envelopes, however, with more environmental parameters that have to be considered and, in addition, a wider range of variation. Environmental effects with a focus on the delamination resistance of FRP laminates for aerospace have been summarised, e.g., by Brunner [9]. Structural composites in service are typically subject to hygro-thermo-mechanical cycles due to considerable variation in the ambient environment under the typical service conditions. The amount and the time-scales of the different factors, e.g., mechanical loads or stresses, temperature, humidity, etc., vary significantly. In aerospace applications of FRP composites, there are additional factors that may further contribute to degradation of the material properties, including their delamination resistance, e.g., particle impact, electromagnetic radiation or lightning [91,92,93,94].

The performance of FRP composites under cryogenic conditions has recently been reviewed by Hohe et al. [95], discussing strength, toughness and failure mechanisms, as well as related material characterisation procedures. Most of the cryogenic toughness data deal with GFRP, indicating a trend for lower fatigue fracture rates at liquid nitrogen temperature (77 K), but some cases of rate increases when temperatures are lowered to liquid helium (4.2 K) are also noted. Clearly, some of the reported results are contradictory. One report on the cryogenic testing of CFRP (IM7/8552) did not find significant reductions in toughness compared with room-temperature, but noted higher toughness at an intermediate temperature [96]. Microscopic investigations show evidence of micro-crack formation at cryogenic temperatures [97,98], e.g., also in cryogenic fuel tanks made from CFRP [99]. These microcracks can provide a path for hydrogen to leak and may contribute to damage initiation and, eventually, lead to failure.

Extensive literature on the fracture testing of FRP composites after or under exposure to different, but constant, environments appears to indicate that most of the test conditions reduce the delamination resistance of the FRP composites. Although changes in toughness or other properties due to a specific factor, e.g., temperature variation, are well understood, even in the space environment, Edwards et al. [92] note that (cite) “*Today, the space environment engineer has a very good understanding of the environmental constituents and has models to predict the flux, fluence, and spatial distributions of each component. The part of the predictive tool that is missing is a thorough understanding of the synergy of how these individual components of the environment interact and produce effect in materials. Often these synergistic effects are not accurately simulated in ground test facilities*”. The statement above refers to conditions in space, but assessing the synergistic effects of other complex service environments essentially presents the same problem. Accelerating ageing under combined exposures and loads is difficult. Often, temperature is chosen as an accelerating factor, or test data are analysed via time–temperature superposition, requiring testing at several different temperature levels. However, for polymer-based composites, the temperature range where an accelerating effect is obtained without activating additional damage mechanisms is limited.

There are a few exceptions noted in the literature, where environmental effects result in observed toughening increases rather than degradation. One example is discussed by Hooper and Subramanian [100]. The absorption of water and of two types of jet fuel by soaking CFRP specimens (equivalent to AS4/3501-6) for about 200 days resulted in weight gains between 0.36 and 2.15%. The quasi-static Mode I toughness increased from about 0.11 kJ/m^2^ (for reference specimens kept in dry condition) to about 0.16–0.17 kJ/m^2^ for the soaked specimens, i.e., by 45–55%. For quasi-static Mode II, the percentage increase was less pronounced, from 0.81 kJ/m^2^ (for dry conditions) to about 0.96–1.02 kJ/m^2^, i.e., by 18–26%. Extensive SEM investigations of the fracture surfaces indicated that the absorption of all fluids changed the fracture surface morphology of Mode I specimens significantly, while for Mode II, only the jet fuel absorption yielded significant morphology changes. Increased cohesive fracture of resin and fibre breaks, as well as fewer fibre–matrix adhesion failures, were identified as likely causes for the observed toughness increase. These effects, however, are in contrast to experiments performed under Mode II fatigue fracture on CFRP reported by Landry et al. [101]. For a CFRP (G40-800/5276-1), an increase in delamination rate for distilled water, hydraulic fluid (Aero-Shell Fluid 41) and de-icing fluid (UCAR ADF XL 54), as well as a decrease in the number of cycles until delamination onset, were noted. The effects were most pronounced for de-icing fluids. A similar decrease under quasi-static Mode I and Mode II loads was observed for CFRP adhesively bonded joints after the application of de-icing fluid [102]. The de-icing fluid was SAFEWAY KF from CLARIANT, diluted with distilled water to concentrations of 2%, 7% and 10%, applied on the surfaces via dip coating (aqueous solution), and then, dried in the oven for 2 h at 40 °C. Then, acclimatisation at room temperature was allowed. For hydraulic aviation fluid MIL-PRF-87257 at a range of different concentrations, a reduction in bond-line strength by 15% and bond-line toughness by 30%, even for low concentrations (3 μg/cm^2^), was noted in Ref. [103]. Another case of partially beneficial environmental exposure on the toughness of FRP was humidity or moisture ingress, which led to plasticisation of the matrix of the CFRP composites. As discussed by LeBlanc and LaPlante [104], in Mixed Mode I/II tests, the Mode I toughness was improved by this, while the Mixed Mode I/II and the Mode II toughness were reduced.

The few examples cited here indicate that the data for environmental effects on delamination resistance, even in seemingly simple cases of one single type of exposure (e.g., a specific fluid) over relatively short times, may yield conflicting results. As soon as different environmental exposures are combined, e.g., temperature and humidity variation (typical for aircraft operation), or temperature with different fluids simultaneously, there is the question of potential synergistic effects of the combined exposures. Of course, this also holds for multiple environmental exposures combined with mechanical loads in typical service conditions.

Synergistic effects in the literature are often noted in the context of improving the fracture properties of CFRP, e.g., in hierarchical composites with nano-fillers (see, e.g., [105,106,107,108]) or in epoxy composites via interpenetrating networks (see, e.g., Farooq et al. [109]). One example of a detrimental synergistic effect, a combined exposure to temperature and humidity, on fatigue delamination onset in bonded joints made with different processes (including one co-cured CFRP composite joint without adhesive) is discussed by Ramirez et al. [110]. A similar result was reported by Tserpes et al. [111] for CFRP joints with intentional pre-bond contamination, representing production problems and in-service bonded repair, respectively. The authors summarise that (cite) “*A combined contamination results in a reduction of the fracture toughness of the bonded jointed that is greater than the reduction caused by each contaminant separately, indicating that a combination of contaminations may be more detrimental to the composite bonded joints’ performance*.” They add that “*In order to evaluate the combined effect of the pre-bond contamination and afterbond exposure to hygrothermal environment on the mode-II fracture toughness of CFRP bonded joints, the contaminated samples underwent aging inside an environmental chamber. Mostly, there was a negative effect of the contamination. Afterbond hygrothermal aging significantly degrades the mode-II fracture toughness of the composite bonded joints. The decrease is larger for the contaminated samples, which reveals that the combined effect is more severe than that of the two effects separately*”.

For GFRP composites with nano- and micro-size-filler-modified epoxy matrix, there is evidence that improving fracture toughness or delamination resistance by adding nano- and micro-scale particles to the epoxy matrix results in the degradation of another property, the compressive strength. Both properties could not be improved simultaneously with the same particle modification of the matrix [112], again indicating the complexity of potential synergistic effects. In view of observed synergistic effects in controlled laboratory tests that improve or degrade the delamination resistance performance of CFRP composites, it seems quite challenging to identify all potential synergistic effects in realistic service environments. Quantifying these sufficiently accurately for establishing predictive models constitutes an even bigger challenge.

There is another aspect that has not been considered yet: the characterisation of, e.g., temperature and moisture effects on the fatigue life of composites is usually performed by first conditioning specimens under constant environments, and then, testing in a different constant environment [113,114,115,116]. However, real structures typically undergo hygro-thermal cycling. Thus, testing in a constant environment, even at temperatures above or below room temperature, e.g., at constant cryogenic temperatures, or in specific chemical environments, e.g., de-icing and cleaning fluids or jet fuel, is not representative of real service conditions and may be non-conservative [116]. Exposure to variable environments and loads, in principle, is possible; however, the effect and extent of detrimental synergistic action of the different factors may depend on the time-scales of the different influencing factors. An important aspect in interpreting the resulting synergistic detrimental effects is the scatter in the experimental data. Developing reliable predictive models, hence, requires test data with low scatter, without losing the scatter due to the typical variability of the CFRP laminates resulting from the respective manufacturing and processing methods.

Clearly, the development of suitable combinations of test methods and modelling or simulation for the prediction of damage accumulation in CFRP— specifically for delamination initiation and propagation under specific service conditions—would be beneficial for structural design with CFRP laminates. In view of all these issues related to current quasi-static fracture or fatigue fracture test methods for CFRP composites, it is not clear which approaches are best suited to providing sufficiently reliable fracture behaviour predictions for CFRP structures and components under service environmental conditions.

### 3.6. FRP Delamination Initiation and Propagation versus Neat Matrix Polymer Toughness

The main mechanisms for microscopic and mesoscopic damage accumulation in FRP composites are matrix cracking, fibre–matrix debonding and fibre breaking. Interlaminar delaminations in FRP have been shown to essentially result from micro- or mesoscopic matrix cracks [117] on a scale of roughly 100–200 μm in diameter. Hence, it could be speculated that the interlaminar delamination resistance of FRP composites is basically governed by the fracture toughness of the matrix polymer between the fibre plies. There are further arguments indicating that Mode I, i.e., the tensile opening load, is possibly the critical stress case [118]. The relationship between neat resin and composite matrix resin toughness is briefly examined via a comparison between the Mode I quasi-static and cyclic fatigue fracture data of a CFRP epoxy laminate and of the respective neat epoxy resin.

If delaminations initiate from fibre–matrix debonding and propagate essentially between fibre plies and the adjacent polymer matrix, the adhesion toughness between the fibre and matrix is the crucial parameter. A priori, this could be higher or lower than the toughness of the polymer matrix. The published data for fibre–matrix adhesion toughness from CFRP epoxy laminates [119] amount to roughly 180-200 J/m^2^, i.e., higher than the typical toughness of neat, unmodified epoxy resins on the order of 70 J/m^2^ [120]. However, the published delamination initiation values of CFRP epoxy composites [9] in the order of 200–400 J/m^2^ tend to be similar or higher than the interfacial toughness. The effective type of delamination propagation, i.e., interlaminar in the matrix or interfacial between matrix and fibre plies, can be verified via post-test microscopic inspection of the fracture surfaces.

**Figure 6:**
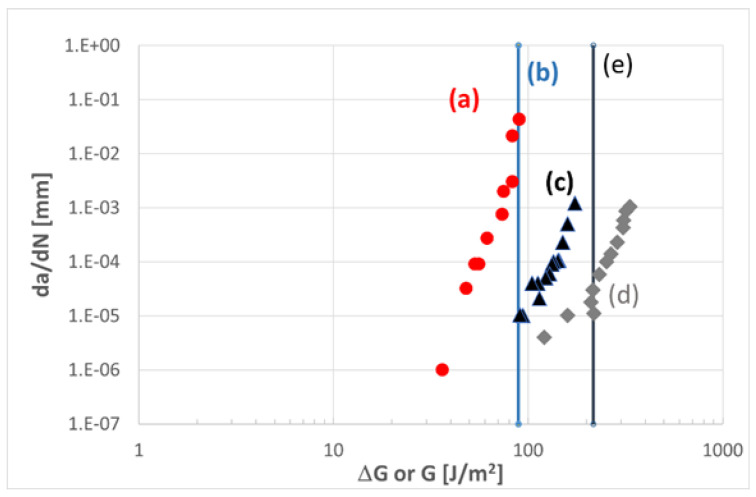
Selected toughness data from the literature: (a) Mode I fatigue fracture of neat RTM6 by Fischer et al. [121], with ΔK converted to ΔG via an average modulus of 3000 MPa; (b) quasi-static Mode I toughness of neat RTM6 from the Hexflow Product Data Sheet [122]; (c) and (d) two sets from Mode I fatigue fracture of a carbon fibre 5HS weave/RTM6 composite by Yutaka Shiino [123], indicating scatter band and testing; and (e) from quasi-static Mode I testing of CFRP RTM6-2 by Wu et al. [124].

One type of CFRP composite with a thermoset matrix, an epoxy, serves as an example of the proposed methodology. Figure 6 summarises the literature data from several sources [121,122,123,124] for a comparison between the quasi-static and cyclic fatigue fracture of neat epoxy (type RTM6) and CFRP made with RTM6 or the equivalent two-component RTM6-2 resin as matrix. The data presented below show that the quasi-static Mode I fracture of neat RTM6 and CFRP with the RTM6 matrix provide upper bounds for the respective Mode I fatigue fracture curves. Further, the neat RTM6 data, both quasi-static and fatigue fracture, provide a lower limit for the Mode I quasi-static and fatigue fracture of CFRP with the RTM6 matrix. Thus, the data suggest that neat polymer toughness and/or fatigue delamination resistance data may provide a lower bound limit for the fracture data of CFRP with the respective polymer as a matrix. However, as discussed by Brunner [125], there may be limitations that have to be considered. One limitation relates to the fact that improvements in the toughness of the neat epoxy resins, e.g., obtained by adding and dispersing nanoparticles, may not fully be transferred to the respective CFRP composite manufactured with such a modified epoxy matrix [126]. In that case, the delamination resistance of the CFRP may be over-estimated by the resin data.

A similar study [127] comparing Mode II measurements of epoxy resin with Mixed Mode I/II delamination resistance of CFRP found a statistically significant relationship between the resin and the CFRP composite data. However, the authors note that (cite) “*The increase in constraint in a composite changes the global behaviour and hugely increases the energy release rate from a bulk, unconstrained state. The results from the five materials presented here suggest that at a local mechanistic level crack behaviour is similar and quantifiable. It has been shown that performing direct measurements of parameters at failure, rather than relying upon shape-function and load-based methods, offers a promising insight into connecting the material behaviour of matrix and composite*”.

Whether neat resin toughness values, either from quasi-static or from cyclic fatigue fracture tests, will provide safe design limits for the CFRP composites with the same resin as a matrix is, therefore, highly speculative. However, it seems feasible in some cases, as exemplified by the case of RTM6 epoxy resin. Nevertheless, there are limitations that have to be explored and sufficiently well understood before this methodology can be proposed as a simpler and less costly approach to obtaining material design data. One question that has not been investigated yet is whether a similar relationship as that observed for epoxy exists between the neat matrix polymer and the composite matrix in the case of thermoplastics. If such relationships are found that allow for the determination of safe design limits, this would significantly reduce the test effort, both in terms of the material used and test time.

Besides polymer-matrix toughening, new processes, specifically different versions of Additive Manufacturing (AM), are extensively discussed in the recent literature (see, e.g., References [3,128,129,130,131]). There are indications that additively manufactured polymer parts may contain more defect sites, e.g., voids or porosity, than parts manufactured by other processes. In long-term service, AM may either yield more crack initiation or a higher probability of crack initiation in comparatively shorter service duration. The question, therefore, is whether analogous multi-site delamination initiation, and then, propagation will also occur in additively manufactured CFRP. There are some reports about relatively weak interlaminar properties of CFRP discussed in the AM reviews cited above. However, it is too early for a conclusive assessment, as noted by Patterson et al. [132] (cite): “*Although it was excluded from the review, one of the most important areas of future research should be on polymer-fiber composites. Some of the studies reviewed during this project discussed fiber-based AM composites but only the information related to the raw material was collected. The effects of the fibers and their placement may have a very large impact on the manufacturing and design approaches needed for AM, particularly FDM and similar processes. A comprehensive review should be done to establish the state-of-the-art, after which specific new research directions can be easily identified*”.

### 3.7. Perspectives for CFRP Composite Structural Design Approaches: Smarter Testing and Beyond

Structural design requires the generation of material data and design allowables for the sizing of the structure. As discussed in Section 2, in aerospace engineering, this is traditionally achieved using the building block or ‘test pyramid’ approach [22]. This procedure is reliable, but also time-consuming and expensive. Thus, there is great interest in the development of so-called ‘smart(er) testing’ approaches, where in essence, some of the building block tests are replaced by models [86]. These approaches are of particular interest for FRP composites due to the difficulty of determining laminate-level properties based only on unidirectional ply properties [133] or basic fibre and resin data [134,135]. Consequently, the state of the art is that each lay-up is treated as a separate material, requiring a separate set of tests to characterise it. The practical implication of this is that designers are limited to a small catalogue of characterised laminates, rather than being able to utilise the full design space, as it is too expensive to generate the needed design allowables. This holds for quasi-static loading, but even more so for fatigue. The hope is that modelling can address this issue, by enabling prediction of the behaviour of an arbitrary lay-up based on a limited set of material input data. In addition, efforts are underway to predict the behaviour of more complex structural elements (e.g., a stiffened panel) based on coupon-level data. This would reduce the amount of testing required at higher levels of the test pyramid. Due to the size of the specimens, these higher-level tests require higher forces, and thus, more expensive test infrastructure. They also require significant investment in the necessary manufacturing tooling to make the specimens, meaning they can only be conducted late in the development process. Reducing the need for such tests through improved modelling thus promises significant costs savings and the validation of structural designs at an earlier stage of the development process [86].

**Figure 7:**
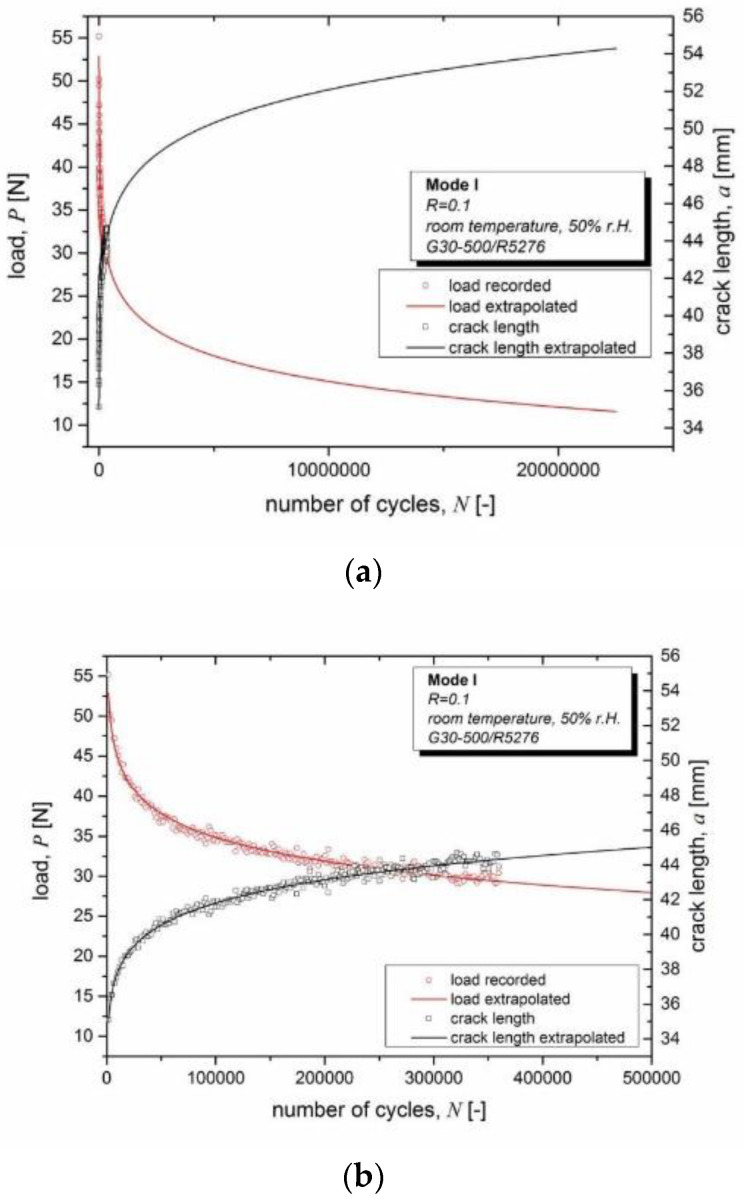
An example of a long-term extrapolation from fitting measured load and crack length data of a Mode I fatigue fracture test, (**a**) full extrapolation, (**b**) showing measured data and extrapolation up to 500,000 cycles.

In order to enable a smarter testing approach, in particular for fatigue, more robust models are thus needed. The key challenges to overcome are, on one hand, reducing the experimental scatter in the data required for the models. The long-term extrapolation of measured raw data over orders of magnitude yields seemingly smooth curves (Figure 7, left panel). Load and crack length values are extrapolated from about 350,000 cycles to more than 20 million cycles, i.e., a factor of 57 in cycle number. This extrapolation, however, assumes that a specific power law fit of the data can be directly extrapolated to any arbitrary number of cycles. If the scatter in the raw data (Figure 7, right panel) is also extrapolated (not shown in Figure), the prediction accuracy is clearly limited. Up to about 350,000 cycles, the scatter in crack length is estimated to be about ±0.5 mm (or ±1% for an average of about 45 mm). The same scatter of ±0.5 mm at a crack length of 35 mm (end of the extrapolation) amounts to about ±3%. For the load, the scatter of ±3 N at 350,000 cycles already amounts to about ±8%, and roughly doubles at the end of the extrapolation. Delamination lengths determined from coupon compliance (displacement divided by load; see, e.g., Stelzer et al. [68,69]), will show effects from the scatter in the load data. Another comparison between extrapolations based on partial data sets for the same CFRP with different numbers of cycles (20%, 40%, 60% and 80% of the full data) with the full data is discussed in detail in Figure 11 on p. 104 of Ref. [69]. It yielded errors of more than 0.6 mm for the 20% fit (extrapolation by a factor of 5 in cycle number), and of more than 0.05 mm for the 40% and 60% fits (extrapolation by a factor of about 2.5 and 1.7, respectively). This at least casts some doubt on the validity of long-term extrapolations by orders of magnitude (i.e., a factor of ten or more).

On the other hand, the question of how to transfer data gathered from one test, to predict the behaviour of a part with a different lay-up, is another as-yet-unsolved challenge. This requires obtaining more insight into the physics and micro-mechanics of composite fatigue, so that one can derive the physical laws that allow such generalisation of test data. Furthermore, it will allow identification of the fundamental material properties that need to be characterised (preferably at the single-ply, or even fibre and matrix levels) in order to be able to predict the behaviour of a full-scale structure. What may help in gaining this understanding is explicit consideration of the fatigue delamination propagation as being the result of an interplay between the amount of work applied in a load cycle and the resistance to crack growth. With this perspective, one could, for example, seek to describe how changing the lay-up affects both (1) how much energy is available for crack growth for a given external load cycle, and (2) how much energy is required to further propagate the delamination. Similar to the argument made in Reference [136] for metals, if one can find the fundamental physical relationships governing these two parameters, one would, in principle, be able to predict the fatigue fracture behaviour of a laminate with any lay-up.

Recently, various types of artificial intelligence (AI), have been applied to the analysis of damage and damage accumulation in FRP composites (see, e.g., [137,138]). AI and related methods may have the potential to find correlations in the interaction between damage present in the FRP composites and the complex environmental and service load effects. This is not limited to material characterisation and analysis, AI may also be applied to structural design [139,140,141,142]. Such investigations yielding clear correlations, in the end, may yield insight into the underlying physical mechanisms and possibly contribute to reducing the experimental scatter, which are both required for robust and reliable predictive models and their validation.

## 4. Summary, Conclusions and Outlook

With respect to the question stated in the title, the authors are convinced that current coupon fracture testing and data analysis are not sufficient to enable reliable predictions of the fatigue delamination initiation and propagation behaviour of CFRP composite components or structures to be made. The specific aspects of coupon testing and analysis are assessed as follows: The effects of fibre bridging in unidirectional fibre-reinforced beam specimens can be accounted for, even though the test procedure and analysis may be rather time-consuming. Thus far, the reproducibility of the procedure has not been quantitatively assessed. Two-dimensional delamination propagation in CFRP composite plates or shells, as examples of structural parts, yields effects that are difficult to predict from the quasi-static or fatigue fracture test coupon data. Laminate lay-up and related fibre bridging seem to play a role in this, as well. The quantification of multiple delaminations has not been solved yet. Even though their initiation and propagation may provide an important toughening effect in CFRP components or structures, predictions require sufficient understanding of the interaction between several delaminations. Possibly, a combination of experiment and modelling will yield more insight. Experimental scatter in material testing limits the precision of input data for modelling or simulation and, thus, directly affects the quality of long-term predictions. The repeatability and reproducibility of the tests so far clearly exceed the variability expected from the measurement resolution specified in the fracture test procedures. Material variability from manufacturing and processing is one factor, and test set-up, test performance and analysis by human operators is another. Effects from the first may possibly be reduced by automated manufacture and processing. The additive manufacturing of CFRP, however, does not yet provide that. Automated data analysis and possibly artificial intelligence for evaluating the data sets may reduce the other effects. Available experimental data on the effects from exposure to different environments mostly indicate decreasing delamination resistance with increasing exposure duration. However, there are noteworthy exceptions yielding improved delamination resistance. Suitable models for predicting the long-term behaviour of delamination initiation and propagation under complex service conditions are still lacking. The same holds for composite parts with complex shapes, or such parts made from hybrid materials. Effects from processing, such as residual stresses and microscopic defects, can play a significant role in determining service life. Whether current non-destructive test methods applied for quality control will indicate all relevant defect types at micro- and mesoscopic scales in CFRP elements or structures for the verification of models is questionable. Quasi-static or fatigue Mode I toughness data from unreinforced polymers used as matrix materials may provide conservative limits for their respective CFRP laminates. How much of a specific toughening effect in the polymer is transferred into the composite when that polymer is used as matrix material has to be evaluated, before quantifying the scatter. Attempts to make the building block design methodology for composite structures more efficient via combinations of experiments and related modelling look promising. Suitable models require input data with sufficiently low scatter, but, even more importantly, they require greater insight into the physics of the damage processes yielding delamination initiation and propagation. Currently, these efforts likely provide the best approach for implementing fracture mechanics data into design for manufacturing weight-optimised and safe damage-tolerant load-bearing composite structures.

## Figures and Tables

**Table 1:** Average errors in blind predictions of residual strength after fatigue loading of the seven models benchmarked in Reference [77].

Load Condition	Average Error (%)
Open-hole tension	
[0/45/90/-45]_2S_ (200 kcycles)	16
[60/0/-60]_3S_ (300 kcycles)	74
[30/60/90/-60/-30]_2S_ (300 kcycles)	26
Open-hole compression	
[0/45/90/-45]_2S_ (200 kcylces)	15
[60/0/-60]_3S_ (300 kcycles)	69
[30/60/90/-60/-30]_2S_ (300 kcycles)	39

## Data Availability

Not applicable.
